# The thioredoxin system protects PSI from photoinhibition in coordination with PSI cyclic electron transport under fluctuating light conditions

**DOI:** 10.1093/pcp/pcaf172

**Published:** 2025-12-23

**Authors:** Yuki Okegawa, Wataru Sakamoto

**Affiliations:** Institute of Plant Science and Resources, Okayama University, Chuo 2-20-1, Kurashiki, Okayama 710-0046, Japan; Institute of Plant Science and Resources, Okayama University, Chuo 2-20-1, Kurashiki, Okayama 710-0046, Japan

**Keywords:** chloroplast, photoinhibition, PSI cyclic electron transport, redox regulation, thioredoxin

## Abstract

Under natural growth conditions, plants are constantly exposed to changes in light intensity. PROTON GRADIENT REGULATION 5 (PGR5)-dependent photosystem I cyclic electron transport (PSI-CET) is known to be required to protect PSI from photoinhibition during such fluctuating light. Moreover, the *Arabidopsis thaliana* (Arabidopsis) *pgr5* mutant cannot survive under fluctuating light conditions due to severe PSI photoinhibition. Recently, we demonstrated that the thioredoxin (Trx) system also supports PSI photoprotection under fluctuating light conditions. Of the five Trx types in Arabidopsis chloroplasts, the *x*- and *y*-type Trxs prevent over-reduction of the PSI acceptor side. In the mutant lacking both types (*trx x trx y1y2*), strong acceptor-side limitation and PSI photoinhibition occurred. To further clarify the roles of PSI-CET and the Trx system in PSI photoprotection, we analyzed multiple mutants. In the *pgr5-5 trx x trx y1y2* quadruple mutant, the PSI acceptor side was more reduced than in either the *pgr5-5* or *trx x trx y1y2* mutant. After exposure to fluctuating light, the *pgr5-5 trx x trx y1y2* mutant also showed more severe PSI photoinhibition. Furthermore, when plants were transferred from constant light to fluctuating light conditions for growth, the *pgr5-5 trx x trx y1y2* mutant displayed pronounced photoinhibition, and its leaves bleached and died. These results indicate that the Trx system acts cooperatively with PSI-CET to protect PSI from photoinhibition under fluctuating light conditions.

## Introduction

Light stress occurs when plants absorb more light energy than their photosynthetic capacity can process. In natural environments, plants frequently encounter fluctuations in both light intensity and quality, making them susceptible to light stress. To cope with such light stress, plants have evolved photoprotective mechanisms that minimize photoinhibition and optimize photosynthesis. These mechanisms encompass both short-term and long-term responses. Short-term adaptations include thermal dissipation of the excess light energy absorbed by photosystem II (PSII) antennae and the suppression of photosynthetic electron transport through downregulation of the cytochrome *b*_6_*f* complex activity, a process known as photosynthetic control (Stiehl and Witt 1969, Horton et al. 1996). Both mechanisms are triggered by acidification of the thylakoid lumen.

In linear electron transport, electrons generated from water splitting at PSII are transferred to NADP^+^ via the cytochrome *b*_6_*f* complex and photosystem I (PSI), resulting in the production of NADPH. This electron transport is coupled to proton translocation across the thylakoid membrane, thereby generating a proton gradient (ΔpH). The ΔpH not only drives adenosine triphosphate (ATP) synthesis but is also crucial for activating photoprotective mechanisms. In contrast, PSI cyclic electron transport (PSI-CET) operates solely through PSI and generates ΔpH without producing NADPH. This additional ΔpH formation supports ATP synthesis and plays a key role in photoprotection (Chaux et al. 2015, Yamamoto and Shikanai 2019). In angiosperms, PSI-CET proceeds through two partially redundant pathways, i.e. the PROTON GRADIENT REGULATION 5 (PGR5)-dependent pathway and the NADH dehydrogenase-like (NDH) complex-dependent pathway (Munekage et al. 2002, Munekage et al. 2004, DalCorso et al. 2008). Both are believed to act as ferredoxin (Fd)-dependent plastoquinone (PQ) reductases, recycling electrons from Fd to the PQ pool, although the details of the PGR5-dependent pathway remain debated (Yamamoto et al. 2011, Hertle et al. 2013, Ruhle et al. 2021). In *Arabidopsis thaliana* (Arabidopsis), the PGR5-dependent pathway is the major PSI-CET route and is required for both induction of thermal dissipation and photosynthetic control (Chaux et al. 2015, Yamamoto and Shikanai 2019). Thermal dissipation is monitored as an energization-dependent (*q*E) component of non-photochemical quenching (NPQ) of chlorophyll fluorescence (Niyogi et al. 1998). In contrast, photosynthetic control can be measured as Y(ND), which indicates the fraction of oxidized P700 (P700^+^) (Klughammer and Schreiber 1994). The Arabidopsis *proton gradient regulation 5* (*pgr5*) mutant cannot induce *q*E and shows extremely low Y(ND) even under high light conditions, making it highly sensitive to high light and fluctuating light (Suorsa et al. 2012, Yamamoto and Shikanai 2019). Particularly under fluctuating light conditions, the PGR5-dependent pathway is indispensable for preventing PSI photoinhibition, and the *pgr5* mutant cannot survive (Suorsa et al. 2012, Yamamoto et al. 2016, Yamamoto and Shikanai 2019). The NDH complex-dependent pathway also contributes to PSI photoprotection under fluctuating light (Kono and Terashima 2016, Zhou et al. 2023). For example, the Arabidopsis *chlororespiratory reduction 2* (*crr2*) mutant, which is defective in the NDH activity, shows more severe PSI photoinhibition than the wild-type (WT) under fluctuating light conditions. The NDH complex-dependent PSI-CET is thought to help balance PSI acceptor-side redox status during the low-light phase of fluctuating light, although the mechanism is still unclear.

In addition to PSI-CET, we recently showed that the thioredoxin (Trx) system is also required for protecting PSI from photoinhibition under fluctuating light conditions (Okegawa et al. 2023). In chloroplasts, Trxs receive electrons from Fd via Fd-Trx reductase, and reduce and activate target proteins in a light-dependent manner (Buchanan 2016). The activity of various proteins involved in photosynthesis and metabolic reactions are regulated by Trxs (Geigenberger et al. 2017, Yoshida and Hisabori 2023). Arabidopsis chloroplasts contain ten classical Trx isoforms (*f*1, *f*2, *m*1, *m*2, *m*3, *m*4, *x*, *y*1, *y*2, and *z*), which are classified into five types (Buchanan 2016). *f*- and *m*-type Trxs together represent more than 90% of all classical Trx proteins in chloroplasts (Okegawa and Motohashi 2015). Previous *in vivo* and *in vitro* studies showed that these Trxs mainly regulate photosynthetic enzymes, including those of the Calvin-Benson-Bassham (CBB) cycle (Geigenberger et al. 2017, Yoshida and Hisabori 2023). In contrast, *x*- and *y*-type Trxs account for only 6.3% and 1.3% of the total, respectively, and have been proposed to function mainly as electron donors to antioxidant enzymes (Collin et al. 2003, Collin et al. 2004, Geigenberger et al. 2017). Their physiological importance, however, remained unclear until recently. We analyzed the *trx x* single, *trx y1y2* double, and *trx x trx y1y2* triple mutants grown under fluctuating light conditions and found that the *trx x* and *trx x trx y1y2* mutants showed severe growth defects (Okegawa et al. 2023). When measured under fluctuating light, PSI acceptor-side limitation was pronounced in the *trx x* and *trx x trx y1y2* mutants. As a result of a short-term fluctuating light treatment, these mutants exhibited stronger PSI photoinhibition than WT. From these results, we concluded that *x*- and *y*-type Trxs are necessary for protecting PSI from photoinhibition under fluctuating light. Nevertheless, the detailed Trx-mediated PSI photoprotection mechanism and its relationship with PSI-CET remain unresolved.

Here, we examined the *crr2-2 trx x trx y1y2* and *pgr5-5 trx x trx y1y2* quadruple mutants to determine how *x*- and *y*-type Trxs and PSI-CET act in PSI photoprotection under fluctuating light conditions. In both quadruple mutants, PSI acceptor-side limitation was more pronounced than in the *crr2-2*, *pgr5-5*, or *trx x trx y1y2* mutants. Furthermore, the *pgr5-5 trx x trx y1y2* mutant showed more severe PSI photoinhibition following brief fluctuating light treatment, which led to marked growth defects during prolonged cultivation under fluctuating light. These findings suggest that *x*- and *y*-type Trxs cooperate with PSI-CET in PSI photoprotection.

## Results

### Combination of *crr2-2* or *pgr5-5* with the *trx x trx y1y2* multiple mutations did not affect plant growth under constant light conditions

**Figure 1:**
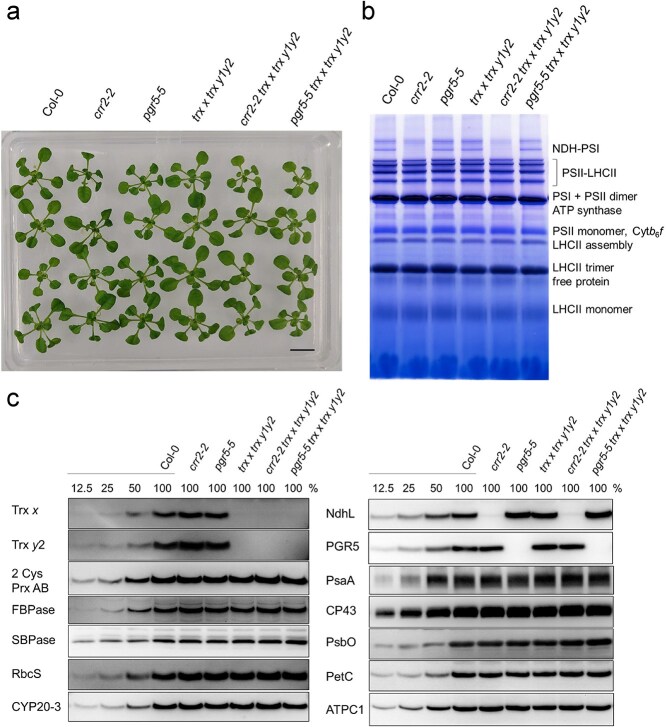
Plant growth and protein accumulation in WT Col-0, *crr2-2*, *pgr5-5*, *trx x trx y1y2*, *crr2-2 trx x trx y1y2*, and *pgr5-5 trx x trx y1y2* plants. (a) Representative photographs of plants. Bar = 1 cm. Plants were grown in chambers under continuous light (50–60 *μ*mol photons m^−2^ s^−1^, 23°C) for 17 days. (b) BN-PAGE analysis of thylakoid protein complexes. The gel was stained with Coomassie Brilliant Blue. The positions of photosynthetic complexes are indicated on the right. (c) Immunoblot analysis of stromal fractions (left) and thylakoid membranes (right). Antibodies used are shown on the left. Protein samples corresponding to 1.0 *μ*g chlorophyll were loaded in each lane.

To investigate the functional interaction of PSI-CET with *x*- and *y*-type Trxs in the photoprotection of PSI, we generated the *crr2-2 trx x trx y1y2* and *pgr5-5 trx x trx y1y2* mutants via crossing. We then grew WT Col-0 and each single, triple, and quadruple mutant under continuous light at 50–60 *μ*mol photons m^−2^ s^−1^ for 17 days (Fig. 1a). All mutants showed growth similar to WT in both fresh weight and chlorophyll content (Table 1). Blue native-polyacrylamide gel electrophoresis (BN-PAGE) indicated that accumulation of major thylakoid complexes (PSII, PSI, ATP synthase) was unaffected in all mutants (Fig. 1b). Consistent with earlier results, PSI-NDH complexes were absent in the *crr2-2* mutant background. Stromal protein levels were also comparable between WT and mutants (Fig. 1c). Unlike the *pgr5-1* mutant, which has often been used as a PGR5-deficient mutant (Munekage et al. 2002), PGR5 protein did not accumulate in the *pgr5-5* mutant (Fig. 1c; Kobayashi et al. 2024).

**Table 1:** Growth phenotypes and *F*_v_/*F*_m_ of WT Col-0, *crr2-2*, *pgr5-5*, *trx x trx y1y2*, *crr2-2 trx x trx y1y2*, and *pgr5-5 trx x trx y1y2* mutant plants under constant light conditions

	Fresh weight (mg)	Chlorophyll content (*μ*g mg^−1^)	*F*_v_ */F*_m_
Col-0	58.9 ± 10.8^a^	2.21 ± 0.13^a^	0.822 ± 0.011^a^
*crr2-2*	59.0 ± 10.0^a^	2.25 ± 0.25^a^	0.828 ± 0.010^a^
*pgr5-5*	57.0 ± 10.4^a^	2.32 ± 0.20^a^	0.819 ± 0.011^a^
*trx x trx y1y2*	57.2 ± 11.6^a^	2.30 ± 0.23^a^	0.825 ± 0.013^a^
*crr2-2 trx x trx y1y2*	60.1 ± 9.9^a^	2.04 ± 0.10^a^	0.826 ± 0.013^a^
*pgr5-5 trx x trx y1y2*	55.1 ± 7.5^a^	2.08 ± 0.05^a^	0.822 ± 0.013^a^

Seedlings were grown under constant light conditions (50–60 *μ*mol photons m^−2^ s^−1^) for 21 days. Each value represents the mean ± SD of ten independent plants. Chlorophyll content was measured per unit fresh weight, with values given as the mean ± SD of three independent replicates. The maximum quantum yield of PSII (*F*_v_*/F*_m_) is shown as the mean ± SD of ten replicates. Means with the same letters are not significantly different among genotypes according to Tukey’s test (*P* < 0.05).

### The *pgr5-5 trx x trx y1y2* quadruple mutant showed more decreased Y(II) and Y(I) than the *pgr5-5* mutant during steady-state photosynthesis

**Figure 2:**
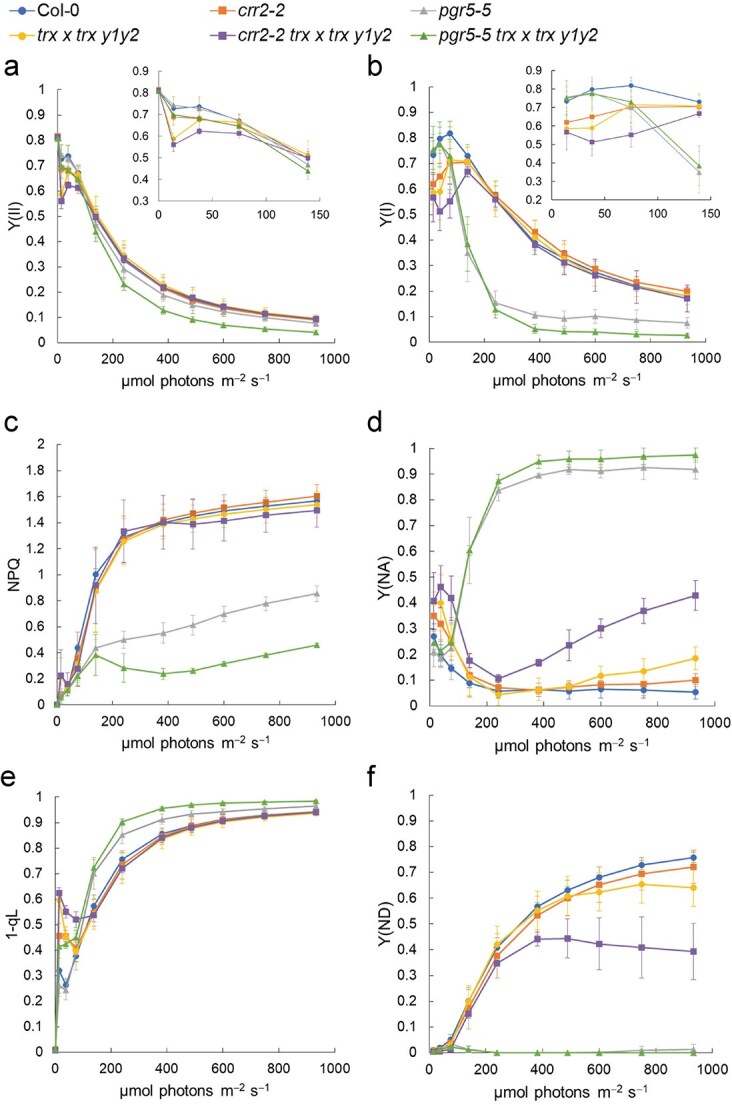
Light intensity dependence of PSII and PSI photosynthetic parameters in WT Col-0, *crr2-2*, *pgr5-5*, *trx x trx y1y2*, *crr2-2 trx x trx y1y2*, and *pgr5-5 trx x trx y1y2* mutants. Chlorophyll fluorescence and P700 parameters were measured during steady-state photosynthesis. (a) Effective quantum yield of PSII [Y(II)]. (b) Photochemical quantum yield of PSI [Y(I)]. (c) The NPQ of chlorophyll fluorescence. (d) Acceptor-side limitation of PSI [Y(NA)]. (e) Reduction level of the PQ pool (1–qL). (f) Donor-side limitation of PSI [Y(ND)]. Insets in (a) and (b) show magnified views of the low light intensities. Each value represents the mean ± standard deviation (SD) of three independent replicates.

To evaluate steady-state photosynthesis in *crr2-2 trx x trx y1y2* and *pgr5-5 trx x trx y1y2*, we measured light-intensity dependence of chlorophyll fluorescence and P700 absorbance with a Dual-PAM system (Fig. 2). The maximum quantum yield of PSII (*F*_v_/*F*_m_), reflecting PSII functionality, did not differ among the six genotypes (Table 1). As reported previously (Kobayashi et al. 2024), the effective quantum yield of PSII [Y(II)] was slightly lower in *pgr5-5* than in WT at light intensities above 100 *μ*mol photons m^−2^ s^−1^ (Fig. 2a). Unlike Y(II), the photochemical quantum yield of PSI [Y(I)] was severely impaired in *pgr5-5* at higher light intensities (Fig. 2b). In contrast, *trx x trx y1y2* resembled WT at higher light intensities, but showed decreased Y(II) and Y(I) at lower light intensities (Fig. 2a and b; insets). A similar pattern to *pgr5-5* was observed in *pgr5-5 trx x trx y1y2*. At lower light intensities, Y(II) and Y(I) were comparable to those in WT, but at higher light intensities, both parameters were lower than in WT and even lower than in *pgr5-5* (Fig. 2a and b). Consistently, *pgr5-5 trx x trx y1y2* showed lower NPQ than *pgr5-5* under high light conditions (Fig. 2c), indicating stronger suppression of luminal acidification required for NPQ induction.

The Y(NA) parameter represents the acceptor-side limitation of PSI, which indicates the fraction of reduced P700. Notably, it was significantly higher in *pgr5-5* than in WT above 100 *μ*mol photons m^−2^ s^−1^, and was even higher in *pgr5-5 trx x trx y1y2* (Fig. 2d). Similarly, the 1–qL, which estimates the reduction level of the PQ pool (Kramer et al. 2004), was further elevated in *pgr5-5 trx x trx y1y2* compared to *pgr5-5* (Fig. 2e). These results indicate that the entire electron transport chain is more reduced in *pgr5-5 trx x trx y1y2* than in *pgr5-5*. The Y(ND) parameter, indicating the fraction of oxidized P700 (P700^+^) in the light, is often used to monitor the photosynthetic control. Since *pgr5-5* fails to induce photosynthetic control due to loss of PSI-CET-dependent luminal acidification, Y(ND) was not induced in *pgr5-5*, and similarly, *pgr5 trx x trx y1y2* also showed no induction of Y(ND) (Fig. 2f). Interestingly, a combination of *crr2-2* and *trx x trx y1y2* mutations affected Y(ND) induction at higher light intensities. As a result, higher Y(NA) was observed in the *crr2-2 trx x trx y1y2* mutant, but PQ reduction (1—qL), Y(II), and Y(I) remained comparable to those in WT under high light.

### PSI and PSII parameters were further impaired in the *crr2-2 trx x trx y1y2* and *pgr5-5 trx x trx y1y2* mutants when measured under fluctuating light conditions

**Figure 3:**
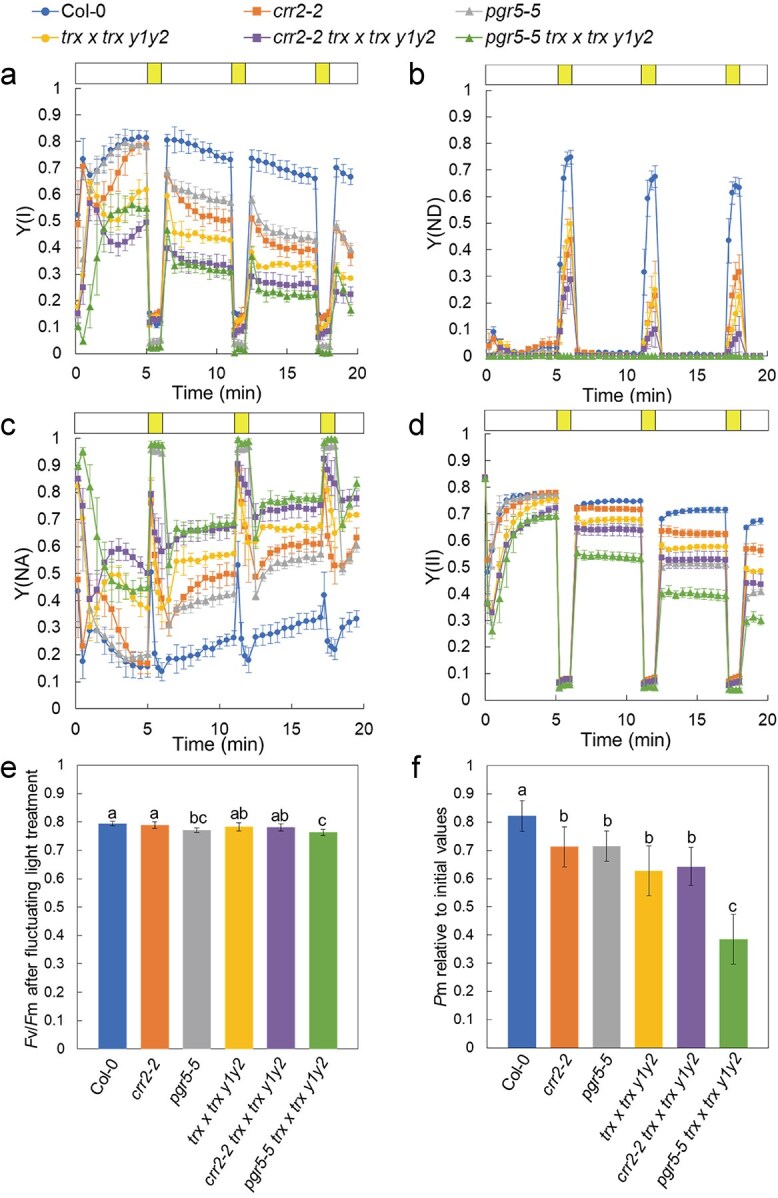
Chlorophyll fluorescence and P700 parameters under fluctuating light in WT Col-0, *crr2-2*, *pgr5-5*, *trx x trx y1y2*, *crr2-2 trx x trx y1y2*, and *pgr5-5 trx x trx y1y2* mutants. Four-week-old plants grown under long-day conditions were subjected to alternating cycles of 5 min low light (white bar, 54 *μ*mol photons m^−2^ s^−1^) and 1 min high light (yellow bar, 1455 *μ*mol photons m^−2^ s^−1^). (a) to (d) represent Y(I), Y(ND), Y(NA), and Y(II), respectively. Each data point is shown as the mean ± SD (*n* = 5 independent plants). (e) PSII photoinhibition. The *F*_v_/*F*_m_ values were measured after fluctuating light treatment. (f) PSI photoinhibition. The *P*_m_ values were compared before and after fluctuating light treatment. Values after fluctuating light exposure were calculated as relative ratios to the pre-treatment values (post/pre). Each value is the mean *±* SD (*n* = 10 independent plants). Different letters indicate statistical differences confirmed by Tukey–Kramer test (*P* < 0.05).

To examine the effects of both PSI-CET and *x*- and *y*-type Trx deficiencies on PSI photoprotection, we analyzed photosynthetic parameters under artificial fluctuating light conditions, which are known to cause stronger photoinhibition of PSI than PSII (Yamamoto et al. 2016). Four-week-old plants grown under long-day conditions were used. The fluctuating light regime consisted of a 5-min low-light phase at 54 *μ*mol photons m^−2^ s^−1^ followed by a 1-min high-light phase at 1455 *μ*mol photons m^−2^ s^−1^, repeated three times (Fig. 3). As reported previously (Yamamoto et al. 2016, Zhou et al. 2023), *crr2-2* and *pgr5-5* were sensitive to fluctuating light, and the quantum yield of PSI was decreased steeply (Fig. 3a). In contrast, Y(I) in *trx x trx y1y2* was already lower than WT during the first low-light phase and declined further with repeated high-light exposure. In both *crr2-2 trx x trx y1y2* and *pgr5-5 trx x trx y1y2*, Y(I) was even lower than in *crr2-2*, *pgr5-5*, and *trx x trx y1y2*, and the values were nearly identical between two quadruple mutants during the final low-light phase. During the high-light phase, higher Y(ND) was induced in WT (Fig. 3b), indicating induction of strong photosynthetic control. Consistent with the results of steady-state photosynthesis (Fig. 2), Y(ND) was not induced at all in *pgr5-5* and *pgr5-5 trx y1y2* during fluctuating light treatment. Suppression of Y(ND) induction was also observed in *crr2-2* and *trx x trx y1y2*, and the Y(ND) value was even lower in *crr2-2 trx x trx y1y2*. Consequently, the Y(NA) of the *crr2-2 trx x trx y1y2* and *pgr5-5 trx x trx y1y2* was higher than in *crr2-2*, *pgr5-5* and *trx x trx y1y2* during both low- and high-light phases (Fig. 3c). Taken together, these results indicate that the PSI electron acceptor side is more reduced in both quadruple mutants.

The impact of fluctuating light on Y(II) differed from that on Y(I). In all genotypes, the decrease in Y(II) caused by fluctuating light was not substantial compared to that observed in Y(I) (Fig. 3d). Among them, the *pgr5-5* mutation caused a greater decrease. Accordingly, Y(II) was lower in *pgr5-5 trx x trx y1y2* than in *crr2-2 trx x trx y1y2*. Consistently, 1-qL was highest in *pgr5-5 trx x trx y1y2*, reflecting over-reduction of the PQ pool (Supplementary Fig. S1a). Unlike during steady-state photosynthesis, transient NPQ was partially induced in both *pgr5-5* and *pgr5-5 trx x trx y1y2* during high-light phase (Supplementary Fig. S1b). This transient induction of qE is likely caused by a temporary imbalance between ΔpH formation and its relaxation.

After measurements under fluctuating light using a Dual-PAM-100 (Fig. 3a–d), we evaluated photoinhibition of PSII and PSI by determining *F*_v_/*F*_m_ and *P*_m_ (the oxidizable fraction of P700), respectively (Fig. 3e and f). Under constant light, *F*_v_/*F*_m_ did not differ among genotypes, whereas fluctuating light slightly decreased *F*_v_/*F*_m_ in the *pgr5-5* background (Table 1 and Fig. 3e). In contrast, severe PSI photoinhibition occurred with fluctuating light treatment (Fig. 3f). To evaluate the impact of fluctuating light on PSI, post-treatment values were normalized to the corresponding pre-treatment values. PSI photoinhibition was more severe in *crr2-2*, *pgr5-5*, and *trx x trx y1y2* than in WT. Combining *crr2-2* with *trx x trx y1y2* did not further exacerbate PSI photoinhibition, although photosynthetic parameters were affected under fluctuating light (Fig. 3a–d). In contrast, *pgr5-5 trx x trx y1y2* exhibited a more pronounced decline in *P*_m_, reaching 0.38 ± 0.09 relative to the pre-treatment level, compared to 0.72 ± 0.05 in *pgr5-5* and 0.63 ± 0.09 in *trx x trx y1y2*, respectively. These results suggest that *x*- and *y*-type Trxs cooperate with the PGR5-dependent pathway to protect PSI from photoinhibition under fluctuating light conditions.

### The *pgr5-5 trx x trx y1y2* quadruple mutant showed severe growth defects when shifted from constant to fluctuating light

**Figure 4:**
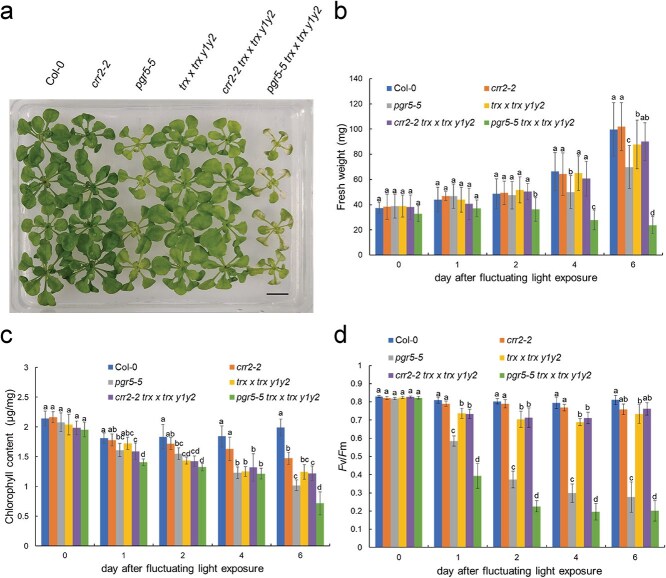
Phenotypic and photosynthetic responses of WT Col-0, *crr2-2*, *pgr5-5*, *trx x trx y1y2*, *crr2-2 trx x trx y1y2*, and *pgr5-5 trx x trx y1y2* plants to fluctuating light. (a) Seedlings were grown under constant light conditions (50–60 *μ*mol photons m^−2^ s^−1^) for 17 days and then transferred to fluctuating light consisting of light cycles of 5 min at 30 *μ*mol photons m^−2^ s^−1^ and 1 min at 500 *μ*mol photons m^−2^ s^−1^ for 6 days. Bar = 1 cm. (b) Fresh weight of seedlings measured before fluctuating light treatment and after 1–6 days of exposure. Each value represents as the mean ± SD (*n* = 10 independent plants). (c) Chlorophyll content per unit fresh weight in seedlings exposed to fluctuating light. Each value is the mean ± SD of three independent replicates. Different letters indicate statistical differences confirmed by Tukey–Kramer test (*P* < 0.05). (d) The maximum quantum yield of PSII (*F*_v_*/F*_m_) before and after 1–6 days of fluctuating light treatment, determined using a two-dimensional fluorescence imager (Closed FluoroCam). Each value represents as the mean ± SD (*n* = 10 independent plants).

To examine the impact of PSI-CET and Trx deficiencies on plant growth under fluctuating light, plants were first grown under constant light conditions (50–60 *μ*mol photons m^−2^ s^−1^) for 17 days. They were then transferred to fluctuating light cycles (5 min at 30 *μ*mol photons m^−2^ s^−1^ and 1 min at 500 *μ*mol photons m^−2^ s^−1^) for an additional 6 days (Fig. 4a). Because the *pgr5-5* mutant cannot survive when grown from germination under fluctuating light conditions (Suorsa et al. 2012), all plants were pre-grown under constant light conditions. After the transfer, all genotypes except *pgr5-5 trx x trx y1y2* showed gradual growth (Fig. 4b). However, the fresh weight of *pgr5-5 trx x trxy1y2* began to decrease after four days of exposure to fluctuating light. In contrast, chlorophyll content declined in all genotypes (Fig. 4c), with the decrease particularly pronounced in *pgr5-5 trx x trx y1y2*, consistent with the fresh weight results. We also measured *F*_v_/*F*_m_ (Fig. 4d). In *pgr5-5* and *pgr5-5 trx x trx y1y2*, *F*_v_*/F*_m_ gradually declined with increasing treatment duration, with the effect being more severe in the quadruple mutant. Although *F*_v_/*F*_m_ was lower in *trx x trx y1y2* and *crr2-2 trx x trx y1y2* than in WT, the decline was not as marked as in lines with the *pgr5-5* mutant background.

After completing six days of fluctuating light treatment, plants were returned to constant light conditions to assess recovery. However, *pgr5-5 trx x trx y1y2* did not recover: leaves bleached completely, and chlorophyll fluorescence could no longer be measured (Supplementary Fig. S2). In contrast, *pgr5-5* leaves remained pale and showed only slight *F*_v_*/F*_m_ recovery. In the other genotypes, *F*_v_*/F*_m_ recovered to levels nearly identical to pre-treatment values (Supplementary Fig. S2b). Overall, these results indicated that both PGR5-dependent PSI-CET and *x*- and *y*-type Trxs are indispensable for sustaining plant growth under fluctuating light conditions.

## Discussion

The *crr2-2* mutant shows photosynthetic parameters similar to WT during steady-state photosynthesis (Fig. 2; Hashimoto et al. 2003). The role of the NDH complex-dependent pathway becomes apparent in the *pgr5* mutant background (Munekage et al. 2004). Introduction of the *crr2* mutation into the *pgr5* mutation further decreased photosynthesis and caused growth defects even under very low light conditions (Munekage et al. 2004, Kobayashi et al. 2024). Similarly, lack of Trx *f* impaired plant growth and photosynthesis in the *pgr5-1* mutant background under normal long-day conditions (Okegawa et al. 2020), although the Trx *f*-deficient *trx f1f2* mutant itself did not show any visible growth differences from WT (Yoshida et al. 2015, Naranjo et al. 2016). Trx *f* catalyzes light-dependent activation of the CBB enzymes such as fructose-1,6-bisphosphatase (FBPase) and sedoheptulose-1,7-bisphosphatase (SBPase; Supplementary Fig. S3). In *trx f1f2*, activation of these enzymes was strongly suppressed (Yoshida et al. 2015, Naranjo et al. 2016). In the *pgr5-1 trx f1f2* mutant, over-reduction of the PSI acceptor side and strong inhibition of photosynthetic initiation were observed during the induction of photosynthesis (Okegawa et al. 2020). In contrast, the *pgr5-5 trx x trx y1y2* grew similarly to WT under constant light conditions (Fig. 1). In *trx x trx y1y2*, the activation of CBB cycle enzymes is slightly delayed during the induction of photosynthesis; however, unlike in *trx f1f2*, it is not suppressed during steady-state photosynthesis (Okegawa et al. 2023). Furthermore, the difference in the photosynthetic parameters between *pgr5-5* and *pgr5-5 trx x trxy1y2* was evident only under higher light conditions (Fig. 2). Therefore, the plant growth of *pgr5-5 trx x trxy1y2* may not have been affected under the growth conditions used in this experiment.

When plants were transferred from constant light to fluctuating light conditions, chlorophyll content and *F*_v_*/F*_m_ substantially decreased in *pgr5-5* and *pgr5-5 trx x trx y1y2* (Fig. 4c and d), indicating severe photoinhibition of PSII. In contrast, the effect on *F*_v_*/F*_m_ was minimal in *crr2-2*, *trx x trx y1y2*, and *crr2-2 trx x trx y1y2*, whereas chlorophyll content declined significantly compared to WT. Caliandro et al. (2013) demonstrated that a decrease in chlorophyll content under fluctuating light represents an acclimatory response rather than PSII damage. In addition, stronger photo-oxidative stress can further accelerate a decrease in chlorophyll content, amplifying this acclimatory response (Yin and Johnson 2000). In our study, fluctuating light caused more severe PSI photoinhibition in *crr2-2*, *trx x trx y1y2*, and *crr2-2 trx x trx y1y2* than in WT (Fig. 3f). Although *F*_v_*/F*_m_ was largely unaffected, PSI photoinhibition may have exacerbated photo-oxidative stress, leading to the largest decrease in chlorophyll content observed in the mutants as part of an acclimatory response. However, applying a harsher fluctuating light treatment—such as shorter fluctuation intervals or greater differences in light intensity than those used in this study—might also impair PSII function in *crr2-2*, *trx x trx y1y2*, and *crr2-2 trx x trx y1y2*, similar to what was observed in *pgr5-5* and *pgr5-5 trx x trx y1y2*.

During steady-state photosynthesis, Y(ND) values in *crr2-2* and *trx x trx y1y2* were similar to WT (Fig. 2f). However, combining these mutations strongly suppressed Y(ND) induction at higher light intensities, indicating diminished donor-side limitation of PSI. This decreased operation of photosynthetic control resulted in an elevated Y(NA), reflecting higher acceptor-side limitation in *crr2-2 trx x trx y1y2* (Fig. 2d). In contrast, Y(II) parameters were not affected in *crr2-2 trx x trx y1y2* under high light. These results suggest that the NDH complex-dependent pathway and *x*- and *y*-type Trxs also help prevent over-reduction of the PSI acceptor side during steady-state photosynthesis, thereby contributing to PSI photoprotection, even when the PGR5-dependent pathway is functional.

Consistent with a previous report (Zhou et al. 2023), during the high-light phases of fluctuating light, Y(ND) induction was significantly suppressed in *crr2-2* (Fig. 3b). Similarly, *trx x trx y1y2* showed lower Y(ND) than WT. In *crr2-2 trx x trx y1y2*, Y(ND) induction was further suppressed, and consequently, a higher level of Y(NA) was observed (Fig. 3c). Since Y(ND) levels of *crr2-2* and *trx x trx y1y2* were not different from WT during steady-state photosynthesis (Fig. 2f), induction of photosynthetic control may be delayed. These results suggest that the NDH complex-dependent pathway and *x*- and *y*-type Trxs contribute to the rapid induction of photosynthetic control in WT during the high-light exposure, which is required to prevent overreduction of the PSI acceptor side. The Y(NA) level was elevated in the *crr2-2* and *trx x trx y1y2* in the first to fourth low-light phases of fluctuating light, with a more pronounced increase in *crr2-2 trx x trx y1y2* (Fig. 3c). Because Y(ND) was not induced during low-light phases, the lower Y(I) was attributable to higher Y(NA) (Fig. 3a and c). Although insufficient induction of photosynthetic control during high-light phases may affect Y(NA) levels in subsequent low-light phases, the NDH complex-dependent pathway and *x*- and *y*-type Trxs may function to oxidize the PSI acceptor side during the low-light phases.

In both *crr2-2 trx x trx y1y2* and *pgr5-5 trx x trx y1y2*, photosynthetic parameters were more impaired than in *crr2-2*, *pgr5-5*, and *trx x trx y1y2* under fluctuating light (Fig. 3). In contrast, *P*_m_ in *pgr5-5 trx x trx y1y2* was lower than in *pgr5-5* or *trx x trx y1y2*, whereas *P*_m_ in *crr2-2 trx x trx y1y2* did not decline further relative to *crr2-2* or *trx x trx y1y2* (Fig. 3f). Recently, Tiwari et al. (2024) reported that the oxidation capacity of P700 remains unaffected despite partial damage to the PSI FeS cluster, indicating that such damage cannot be detected by measuring the *P*_m_ value. Therefore, *P*_m_ measurements may not reflect differences in the extent of PSI photoinhibition among *crr2-2*, *trx x trx y1y2*, and *crr2-2 trx x trx y1y2*. Nonetheless, our results indicate that both PSI-CET pathways and *x*- and *y*-type Trxs are required for preventing over-reduction of the PSI acceptor side and act cooperatively to protect PSI from photodamage.

In contrast to the cooperative role of *x*- and *y*-type Trxs in PSI photoprotection together with PSI-CET, *m*-type Trx has been proposed to negatively regulate both PSI-CET pathways (Courteille et al. 2013; Supplementary Fig. S3). NDH activity was increased in the Arabidopsis mutant lacking Trx *m*4, although the specific target of Trx *m*4 in the NDH complex-dependent pathway remains unclear. We also demonstrated that Trx *m*4 downregulates the PGR5-dependent pathway by forming a complex with PGRL1 (Okegawa and Motohashi 2020). Although these roles may seem contradictory at first glance, different Trx types may act in concert to support chloroplast redox homeostasis and overall photosynthetic resilience.

Unlike *trx x* and *trx x trx y1y2*, other Trx mutants did not show marked growth defects specifically under fluctuating light (Dziubek et al. 2024). However, the function of Trxs under fluctuating light has been investigated. Trx *f*1 has only a minor effect on light acclimation under fluctuating light conditions, whereas Trx *m*1 and *m*2 are required for full photosynthetic activity during the high-light phases (Thormahlen et al. 2017, Dziubek et al. 2024). Furthermore, a mutant with constitutively active NADP-malate dehydrogenase (NADP-MDH) showed severe growth defects under fluctuating light (Yokochi et al. 2021). NADP-MDH, a key enzyme of the malate valve—a shuttle system that balances the ATP/NAD(P)H ratio—is activated by either *f*- or *m*-type Trx.

In addition to classical Trxs, chloroplasts have another Trx system: NADPH-Trx reductase C (NTRC; Supplementary Fig. S3). NTRC uses NADPH as an electron donor and predominantly reduces 2-Cys peroxiredoxin (2-Cys Prx; Perez-Ruiz et al. 2006; Serrato et al. 2004). Although *ntrc* mutants already show growth defects under constant low light, their growth becomes even worse when shifted to fluctuating light (Dziubek et al. 2024, 2025). In *ntrc*, the reduction of 2-Cys Prx and FBPase was suppressed, resulting in decreased carbon fixation. NTRC has been proposed as a key component of the photoprotective mechanism because 2-Cys Prx eliminates hydrogen peroxide, and NTRC serves as its primary electron donor (Pulido et al. 2010). However, recent findings demonstrate that NTRC does not directly contribute to photoprotection under fluctuating light conditions (Dziubek et al. 2025). Instead, it plays a crucial role in regulating photosynthetic acclimation by maintaining chloroplast redox balance to optimize CBB cycle activity. These results imply that the Trx system central to PSI photoprotection under fluctuating light conditions is *x*- and *y*-type Trxs, whose function we previously described (Okegawa et al. 2023). *x*-type Trx is also the most efficient reductant of 2-Cys Prx among light-dependent Trx isoforms (Collin et al. 2004). However, no difference in the redox levels of 2-Cys Prx was observed between WT and *trx x trx y1y2* under fluctuating light (Okegawa et al. 2023). Consistent with findings from the characterization of the *ntrc* mutant (Dziubek et al. 2025), 2-Cys Prx does not appear to be the primary contributor to photoprotection under fluctuating light. Therefore, identifying alternative target proteins of the *x*- and *y*-type Trxs is necessary to elucidate the Trx-mediated PSI photoprotection mechanism (Supplementary Fig. S3).

## Conclusion

In this study, we demonstrated that combining PSI-CET deficiency with loss of *x*- and *y*-type Trxs further exacerbates PSI photoinhibition under fluctuating light. Specifically, when plants were transferred from constant light to fluctuating light conditions for growth, the *pgr5-5 trx x trx y1y2* mutant failed to recover and ultimately died. These findings confirm that PSI photoprotection is indispensable for plant growth under fluctuating light conditions. Although the importance of *x*- and *y*-type Trxs in PSI photoprotection is becoming clearer, their molecular mechanisms remain largely unknown, particularly with respect to their specific target proteins. Further studies are required to provide deeper insight into the regulatory mechanisms underlying Trx-mediated PSI photoprotection.

## Materials and Methods

### Plant material and growth conditions

Arabidopsis (*A. thaliana*) accession Columbia-0 (Col-0) was used as WT. The *trx x trx y1y2* mutant (Okegawa et al. 2023) is in the Col-0 background, whereas the *crr2-2* (Hashimoto et al. 2003) and *pgr5-5* (Kobayashi et al. 2024) mutants are in the Columbia *gl1* background. To generate quadruple mutants, the *trx x trx y1y2* mutant was crossed with *crr2-2* or *pgr5-5*. In F2 progeny from these crosses, the presence of the T-DNA insertion and respective mutations were confirmed by polymerase chain reaction (PCR) (primers listed in Supplementary Table S1).

Plants were grown either in soil or on Petri dishes containing Murashige and Skoog (MS) medium supplemented with 1.0% (w/v) agar and 1% (w/v) sucrose in growth chambers (50–60 *μ*mol photons m^−2^ s^−1^, 23°C). For fluctuating light treatment, a timer-controlled regime of 5 min at low light (30 *μ*mol photons m^−2^ s^−1^) and 1 min at high light (500 *μ*mol photons m^−2^ s^−1^) was applied.

### Sodium dodecyl sulphate-PAGE (SDS-PAGE) and immunoblot analysis

Chloroplasts were isolated from 3- to 4-week-old leaves, as described previously (Okegawa et al. 2022). Briefly, chloroplasts were ruptured in 25 mM HEPES-KOH (pH 7.6) containing 3 mM MgCl_2_ and centrifuged at 10 000 g for 3 min. The pellet (thylakoid fraction) and soluble protein (stromal fraction) were dissolved separately in SDS-sample buffer. Proteins were then separated by 12.5% or 15% SDS-PAGE using the Laemmli Tris-glycine system (Laemmli 1970) or by 16.5% SDS-PAGE with a Tris-tricine buffer system (for PGR5 detection; Schagger and von Jagow 1987). After electrophoresis, proteins were transferred to PVDF membranes and probed with specific antibodies. Immunoblot signals were detected with Chemi-Lumi One Super (Nacalai, Japan) and imaged using a ChemiDoc XRS+ system (Bio-Rad, USA).

### BN-PAGE analysis

BN-PAGE was performed as described (Shimizu et al. 2008). Freshly isolated thylakoid membranes were gently washed twice with buffer containing 25 mM BisTris-HCl (pH 7.0), 20% glycerol, and then solubilized in the same buffer containing 1% (v/v) β-dodecyl-maltoside. Solubilized membranes were separated by 4%–16% gradient BN-PAGE, and proteins were visualized with Coomassie Brilliant Blue staining.

### *In vivo* measurements of chlorophyll fluorescence and P700 absorption changes

Chlorophyll fluorescence and P700 absorption changes in the PSI reaction center were measured simultaneously using a portable fluorometer (DUAL-PAM-100 [MODULAR version] equipped with a P700 dual-wavelength emitter at 830 and 870 nm; Walz, Germany). Plants were dark-adapted for 30 min before measurement, and detached leaves were used. Minimum fluorescence (*F*_o_) was obtained with a weak measuring light (red, 620 nm, 0.05–0.1 *μ*mol photons m^−2^ s^−1^). A saturating pulse (SP, 300 ms, 10 000 *μ*mol photons m^−2^ s^−1^) was applied to determine maximum fluorescence in the dark-adapted state (*F*_m_) and during actinic light (AL) illumination (*F*_m_′). Steady-state fluorescence (*F*_s_) was recorded during AL illumination (635 nm, 14–933 *μ*mol photons m^−2^ s^−1^). The maximum quantum yields of PSII (*F*_v_/*F*_m_), Y(II), and NPQ were calculated as (*F*_m_ − *F*_o_)/*F*_m_, (*F*_m_′ − *F*_s_)/*F*_m_′, and (*F*_m_ − *F*_m_′)/*F*_m_′, respectively (Genty et al. 1989). qL was calculated according to a previously described protocol (Kramer et al. 2004).

P700 redox changes were monitored as absorbance differences at 830 and 875 nm. *P*_m_ (maximum oxidizable P700) was obtained with an SP in the presence of far-red light (720 nm). The maximal oxidized P700 under AL illumination (*P*_m_′) was determined with an SP. The P700 signal (*P*) was recorded immediately before each SP. Y(I) was calculated as (*P*_m_′ − *P*)/*P*_m_. Y(NA) was calculated as (*P*_m_ − *P*_m_′)/*P*_m_. Y(ND) was calculated as *P*/*P*_m_. These three complementary yields satisfy Y(I) + Y(NA) + Y(ND) = 1 (Klughammer and Schreiber 1994).

To assess fluctuating light effects, leaves were treated using a DUAL-PAM-100 as described (Yamamoto et al. 2016). Leaves were illuminated with 5 min of low light (54 *μ*mol photons m^−2^ s^−1^) and 1 min of high light (1455 *μ*mol photons m^−2^ s^−1^), repeated for three cycles. Four-week-old plants grown under long-day conditions were dark-adapted for 30 min, and 12 mm leaf discs were excised. After initial *F*_v_*/F*_m_ and *P*_m_ measurements, discs were subjected to fluctuating light, and photosynthetic parameters were monitored. After treatment, discs were placed between wet tissue paper, incubated in the dark for 30 min, and *F*_v_*/F*_m_ and *P*_m_ were remeasured. Photoinhibition of PSI was expressed as the relative decrease in *P*_m_, compared to pre-treatment values.

To analyze *F*_v_/*F*_m_ when grown under fluctuating light conditions (Fig. 4), chlorophyll fluorescence was measured with a two-dimensional imager (Closed FluoroCam, Photon Systems Instruments, Brno, Czech Republic). Plants were dark-adapted for 30 min before measurement.

### Analysis of chlorophyll content

Leaves (30 mg fresh weight) were collected from seedlings grown on MS plates and immediately ground in liquid nitrogen. Chlorophyll was extracted in 80% acetone, and content was determined spectrophotometrically as described (Porra et al. 1989).

### Statistical analysis

All calculations were based on at least three independent biological replicates (see Figure legends). Significant differences among samples were determined by Tukey’s multiple comparison tests (*P* < 0.05).

## Supplementary Material

pcp-2025-e-00262-File007_pcaf172

## Data Availability

Sequence data cited in this article are available in the Arabidopsis Information Resource (https://www.arabidopsis.org/) under the following accession numbers: *Trx x* (At1g50320), *Trx y1* (At1g76760), *Trx y2* (At1g43560), *PGR5* (At2g05620), *CRR2* (At3g46790). The authors confirm that all data supporting this study are included in the article and its supplementary materials. Additional datasets are available upon reasonable request to the corresponding author.
