# Supplementary material for: The thioredoxin system protects PSI from photoinhibition in coordination with PSI cyclic electron transport under fluctuating light conditions
Source: Plant Cell Physiol. 2025 Dec 23;67(6):928–42. doi: 10.1093/pcp/pcaf172 (PMC13317958; doi:10.1093/pcp/pcaf172)
Supplement: pcp-2025-e-00262-File007_pcaf172 [file pcp-2025-e-00262-file007_pcaf172.pdf]

Supplementary Fig. S1

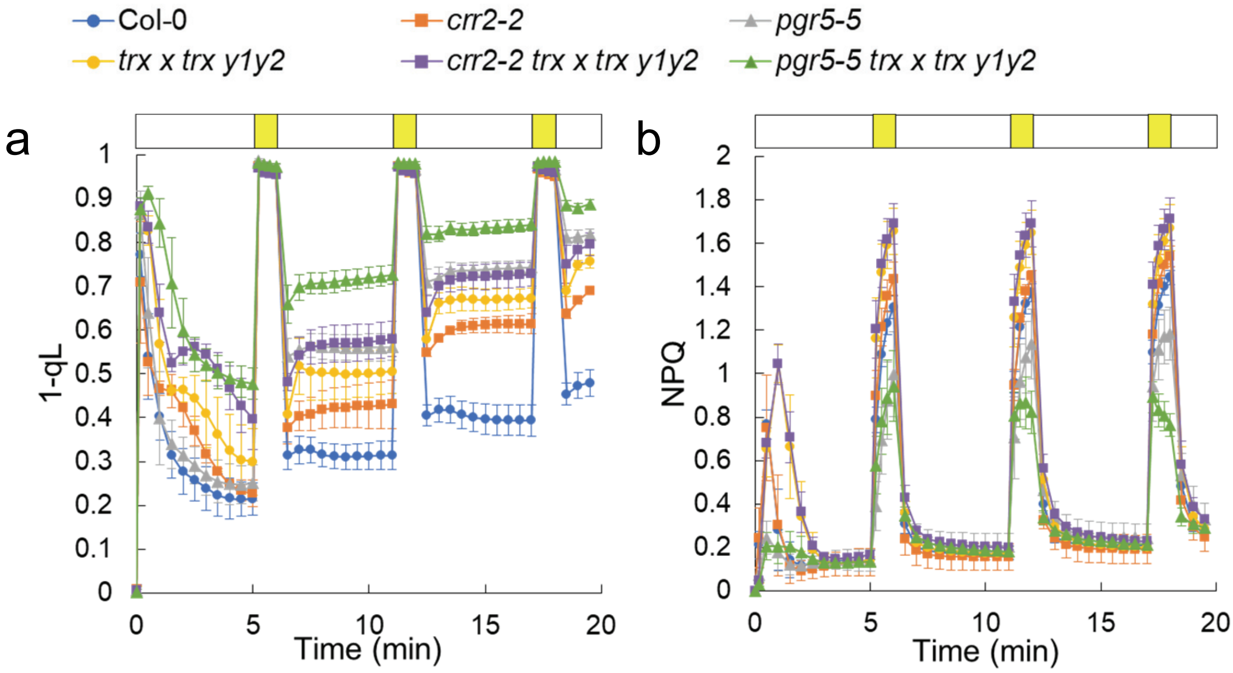

Supplementary Fig. S1 Chlorophyll fluorescence and P700 parameters under fluctuating light in WT Col-0, *crr2-2*, *pgr5-5*, *trx x trx y1y2*, *crr2-2 trx x trx y1y2* and *pgr5-5 trx x trx y1y2* mutants. Four-week-old plants grown under long-day conditions were exposed to cycles of low light (white bar, 54 µmol photons m^{-2} s^{-1}) and high light (yellow bar, 1,455 µmol photons m^{-2} s^{-1}). **(a)** Reduction level of the PQ pool (1 – qL). **(b)** The non-photochemical quenching (NPQ) of chlorophyll fluorescence. Each data point represents the mean ± SD (*n* = 5 independent plants).

Supplementary Fig. S2

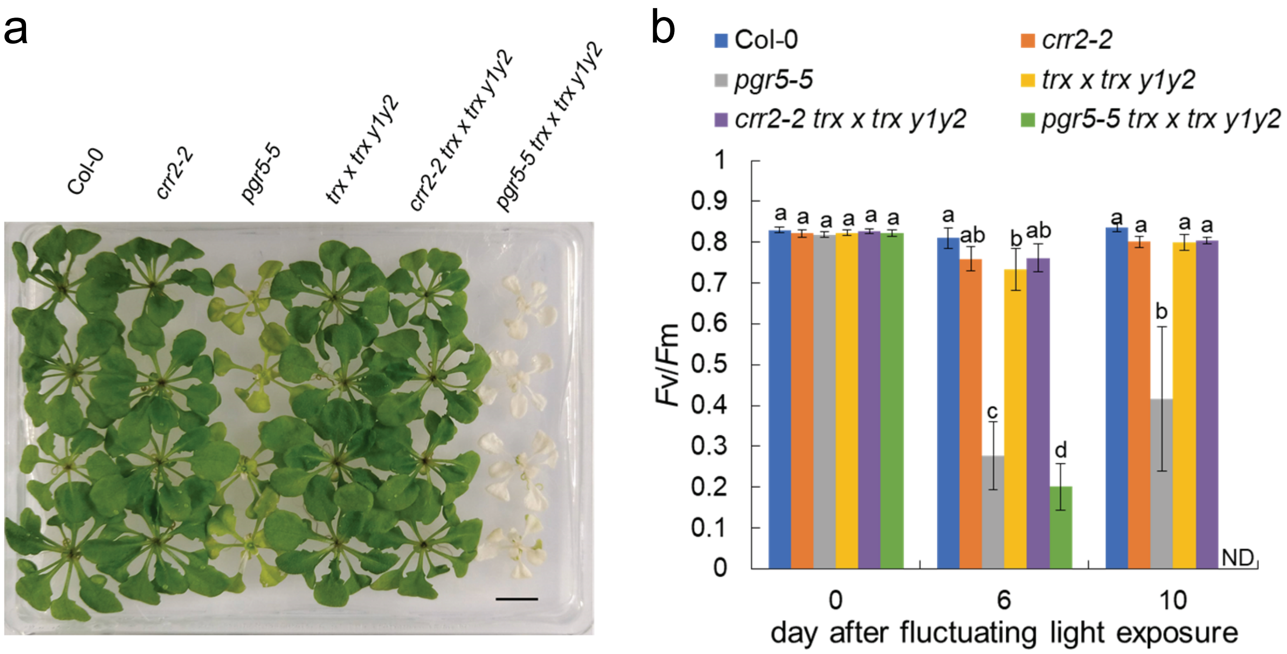

Supplementary Fig. S2 Impact of fluctuating light on growth of WT Col-0, *crr2-2*, *pgr5-5*, *trx x trx y1y2*, *crr2-2 trx x trx y1y2*, and *pgr5-5 trx x trx y1y2* plants. **(a)** Seedlings were grown under constant light (50–60 μmol photons m^{-2} s^{-1}) for 17 days and then transferred to fluctuating light cycles of 5 min at 30 μmol photons m^{-2} s^{-1} and 1 min at 500 μmol photons m^{-2} s^{-1}) for 6 days. They were subsequently returned to constant light for four more days. Bar = 1 cm. **(b)** The maximum quantum yield of PSII (*F*v/*F*m) in seedlings before fluctuating light treatment, 6 days after treatment, and 4 days after recover under constant light conditions, measured with a two-dimensional fluorescence imager (Closed FluoroCam). Each value is the mean ± SD (*n* = 10 independent plants). ND = not detected.

Supplementary Fig. S3

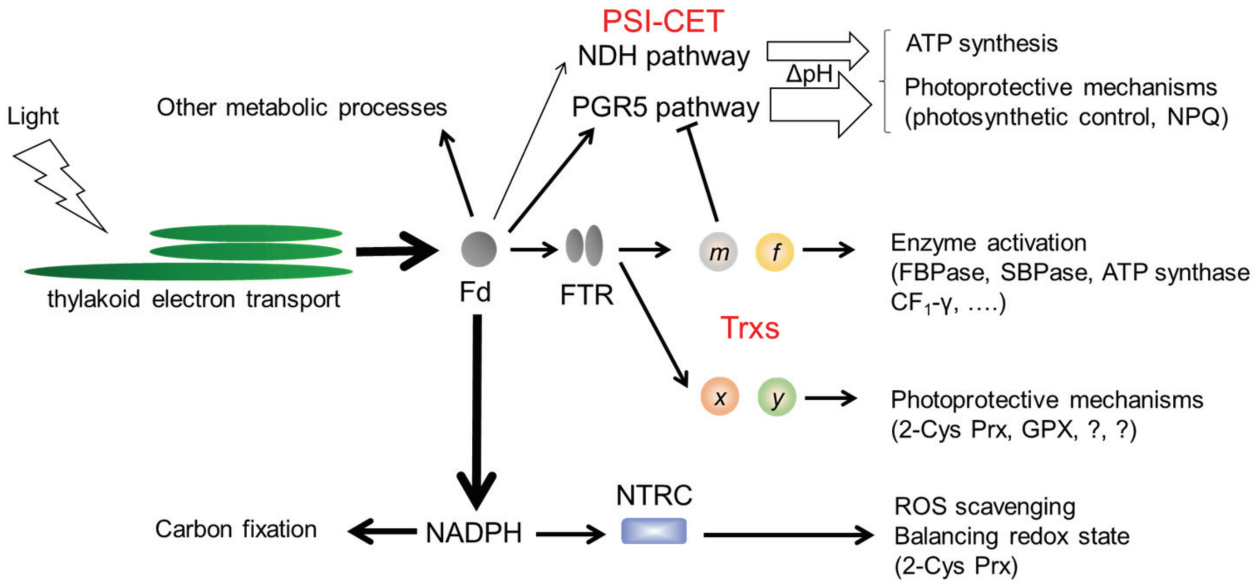

Supplementary Fig. S3 The relationship between PSI-CET pathways and the Trx system Under fluctuating light conditions, PSI-CET and Trx system cooperatively protect PSI from photoinhibition. In chloroplasts, Fd is reduced in a light-dependent manner and donates electrons to a variety of reactions. Both PSI-CET pathways (NDH and PGR5) accept electrons from Fd. In Arabidopsis, PGR5-dependent PSI CET is the major pathway and mainly contributes to ATP synthesis and photoprotective mechanisms. One of the Trx system, Fd/Trx pathway, also depends on Fd for its supply of electrons and regulates many enzymes. *f*- and *m*-type Trxs mainly modulate photosynthesis-related enzymes, including those in the Calvin-Benson-Bassham (CBB) cycle. *m*-type Trx (Trx *m*4) also regulate both PSI-CET. In contrast, *x*- and *y*-type Trxs mainly serve as reducing substrates for antioxidant enzymes, such as 2-Cys Prx and GPX. However, their targets functioning in PSI photoprotection under fluctuating light remains unknown. The other Trx system, NTRC pathway, uses NADPH as electron donors and mainly reduces 2-Cys Prx. Solid arrows indicate the flow of electrons. PSI-CET, Photosystem I cyclic electron transport; Trx, thioredoxin; NDH, NADH dehydrogenase-like complex; PGR5, PROTON GRADIENT REGULATION 5; Fd, ferredoxin; NTRC, NADPH-dependent thioredoxin reductase C; GPX, glutathione peroxidase; 2-Cys Prx, 2-Cys peroxiredoxin.

Supplementary Table S1. Primers used in this study.

The following primers used for genotyping:

Trx *x*-LP	5'-TTTAAACAGGAATGTTGATGCTATG-3'
Trx *x*-RP	5'-CAAAAGGAAGCTCAACTCTAAACAG-3'
Trx *y*1-LP	5'-GGTTCCCCATCTTTGAATAGG-3'
Trx *y*1-RP	5'-AACATGTGGGCCTAGAACATG-3'
Trx *y*2-LP	5'-CGATGGATTAGCGAACTATGC-3'
Trx *y*2-RP	5'-GAGCAAACAATCAACAATGGC-3'
LB (GK)	5'-ATATTGACCATCATACTCATTGC-3'
LBb1.3 (SALK)	5'-ATTTTGCCGATTTCGGAAC-3'
*crr*2-*2*-F (NlaIII)	5'-GAACTATATATGTCTGGAACG-3'
*crr*2-*2*-R (NlaIII)	5'-ATAGTTCCCTGCATTTTTTGG-3'
pgr5-5-F (HhaI)	5'-ATGGCTGCTGCTTCGATTTCTG-3'
pgr5-5-R (HhaI)	5'-GAGAATGGCAGCAGTTATGTGTG-3'
